# Which surgery is ‘best’ for patients with PUJ obstruction in a poorly functioning kidney?

**DOI:** 10.4103/0970-1591.30256

**Published:** 2007

**Authors:** Rob Pickard

**Affiliations:** Department of Urology, Freeman Hospital, Newcastle upon Tyne. NE7 7DN, UK. E-mail: r.s.pickard@ncl.ac.uk

Expert opinion backed by an evidence base of limited quantity and quality would suggest that dismembered pyeloplasty using an open or laparoscopic approach represents optimum treatment for patients with primary PUJ obstruction.[1] For those with impaired function of the affected renal unit, the situation is less clear, with pyeloplasty, endoluminal techniques and nephrectomy all being advocated. To decide which is the best, an adequately powered randomized study would be required with appropriate pre-stated outcome variables, including symptomatic and functional results together with long-term nephrectomy rate. The data obtained would then allow a cost-effectiveness analysis to determine which management strategy gave the best trade-off between patient benefit and cost reduction. In the continued absence of such data, patients will have to rely on the opinion of their clinician, who will take into account the clinical assessment, past surgical experience and relevant information from the literature. Although the current paper[2] scores low on the quality of evidence, it does provide interesting reading that will help shape such opinion. Of particular note are the following: It seems sensible to base treatment on the GFR of the affected kidney rather than the percentage split function, and the symptomatic results of dismembered pyeloplasty are encouraging in the short term. Endoluminal incision was less effective although morbidity and, probably, costs were lower. Objectively functional preservation was achieved in the short term by both techniques, but one would suspect that further deterioration and subsequent nephrectomy would be seen in some patients in the longer term. In summary it is an interesting audit within a single institution, but management decisions for such patients will still have to rely more on the art rather than science of medicine.
